# *In vitro* expansion of human glioblastoma cells at non-physiological oxygen tension irreversibly alters subsequent *in vivo* aggressiveness and AC133 expression

**DOI:** 10.3892/ijo.2011.1271

**Published:** 2011-11-23

**Authors:** ERIKA BOURSEAU-GUILMAIN, LAURENT LEMAIRE, AUDREY GRIVEAU, ERIC HERVOUET, FRANÇOIS VALLETTE, FRANÇOIS BERGER, PHILIPPE MENEI, JEAN-PIERRE BENOIT, DIDIER WION, EMMANUEL GARCION

**Affiliations:** 1 Laboratoire d’Ingénierie de la Vectorisation Particulaire, Inserm, UMR_S 646, Université d’Angers, Angers; 2 Institut des Neurosciences, Inserm, UMR_S 836, Université Joseph Fourier, Grenoble; 3 Département de Neurochirurgie, CHU, Angers; 4 Centre de Recherche en Cancérologie (CRCNA), Inserm, UMR_S 892, Université de Nantes, Nantes, France

**Keywords:** glioblastoma, CD133, tumor niche, cancer stem cell culture, HIF-1α

## Abstract

Among markers of glioblastoma initiating cells, AC133 has been shown to be associated with glioblastoma resistance and malignancy. Recently, it was demonstrated that increasing oxygen tension (pO_2_) down-regulated AC133 expression in glioblastoma cells *in vitro*. In order to better understand extrinsic factor regulation of AC133, this work aimed to investigate the relationship between cell culture pO_2_, AC133 expression, and tumor development and phenotype. Using treatments with CoCl_2_ and HIF-1α shRNA knockdowns on non-sorted human primary glioblastoma cells cultured at low (3%) versus high (21%) oxygen tension, we established a responsibility for low pO_2_ in the maintenance of high levels of AC133 expression, with a major but non-exclusive role for HIF-1α. We also demonstrated that human glioblastoma cells previously cultured under high oxygen tension can lose part of their aggressiveness when orthotopically engrafted in SCID mice or lead to tumors with distinct phenotypes and no re-expression of AC133. These observations showed that the specific pO_2_ microenvironment irreversibly impacts glioblastoma cell phenotypes, highlighting the pertinence of culture conditions when extrapolating data from xenogenic models to human cells in their source environment. They also raised AC133 as a marker of non-exposure to oxygenated areas rather than a marker of aggressiveness or low pO_2_ niches.

## Introduction

Central nervous system glioblastomas are among the most aggressive and treatment-resistant cancers. The recent discovery of self-renewing and uniquely tumorigenic brain tumor stem cells (BTSCs) (1–4), also referred to as brain cancer initiating cells, points to the presumption that this cancer stem cell subpopulation intrinsically resistant to radio and chemotherapeutic treatments might be responsible for the phenotypical derivation of tumors and their recurrence.

Instead of specific markers, makers shared with cancer stem cells (5,6) and BTSCs, such as AC133 (2), CD15 (7), and CD171 (8), have been documented. Among these, AC133, an epitope of the CD133 protein, which is itself a pentaspan glycoprotein identified firstly on hematopoietic stem cells, is the best known (9–12). *In vitro*, in the presence of EGF, FGF-2, and heparin, AC133 expressing cells isolated from human glioblastoma regenerate, form neurosphere-like colonies and are capable of generating cells that express markers of differentiated neural cells (2). In xenograft models using immunodeficient mice, they lead to cancers that are phenotypically similar to the original tumors (2). AC133-positive cancer cells are also particularly resistant to radiotherapy (13) and TRAIL-mediated apoptosis (14). In addition, these cells are capable of promoting tumor neovascularization by producing VEGF (15). Finally, AC133 overexpression in human gliomas is associated with poor clinical outcome (16).

Although these findings are in line with the relevance of developing targeted strategies against BTSCs in glioblastomas through AC133 recognition, other observations argue for a more complex reality. Indeed, while less tumorigenic than their AC133-positive counterparts (3), AC133-negative cells can lead to tumors with a distinct phenotype (17). Moreover, the occurrence of BTSCs does not exclude the existence of cellular networks in which individually non-tumorigenic cell populations might cooperate to produce tumors (18). In addition, the tissue microenvironment might exert pivotal effects for tumor development (18,19), and extrinsic cell modulators may drive the expression of intrinsic markers.

In line with this, AC133 expression in glioblastoma has been associated either with anatomical while not necessarily functional perivascular niches (20) or hypoxic pseudopalissading necrotic regions (21,22). Thus, it is not yet understood whether AC133 incidence in those areas is due to improved survival of AC133-positive cells or positive regulation of AC133 expression. As oxygen is involved in the stem cell behavior (23) and tumor aggressiveness of human glioblastoma (24), it is important to consider its role on AC133 expression and AC133-positive BTSC performance. As such, it has recently been demonstrated that exposure to low oxygen tension (pO_2_) allows for maintenance of the AC133 phenotype of non-sorted human glioblastoma cells *in vitro* (25). In addition, sorted AC133-positive human glioblastoma cells preserve their stem cell phenotype under low oxygen tension *in vitro* (26).

Nonetheless, these studies did not address how cell culture pO_2_ might affect the AC133 phenotype and the behavior of cancer cells following implantation in animals. In our study, we demonstrate that xenogenic experimental tumors, obtained from non-sorted human glioblastoma cells cultured either at 3 or 21% O_2_, can significantly differ. In this context, we investigate whether AC133 is an indicator of low oxygen tension or of tumor aggressiveness. Finally, we discuss our data regarding the relevance of biopsy-derived models for functional investigations or for therapeutic targeting purposes.

## Materials and methods

### Patient tissue samples and human glioma cell cultures

Specimens from patients undergoing biopsy for *de novo* glioma were obtained from the Department of Neurosurgery of the Angers CHU (France), and from the Department of Neurosurgery of the Grenoble CHU (France), with institutional review board approvals. Pathologic diagnosis established that GlioA, GlioB, and GlioC tumor samples were grade IV WHO glioblastomas. Straight after tissue dissociation as previously described (25), cells were plated on uncoated plastic flasks at 2×10^4^/ml of defined medium and cultured at 37°C under an atmosphere containing 5% CO_2_ and either 3 or 21% O_2_. GlioA, GlioB and GlioC were cultured in Dulbecco’s modified Eagle’s medium: Nutrient Mixture F-12 (DMEM/F12, Biowhittaker, Verviers, Belgium) added with Glutamax, B27 and N2 supplements (Invitrogen, Cergy Pontoise, France), recombinant human EGF and FGF-2 (20 ng/ml each, R&D Systems Europe, Lille, France), and heparin (5 μg/ml, Sigma-Aldrich, Lyon, France). Growth factors and supplements were added every 3 days for a period of 10–15 days, until new dissociations with Versene (Lonza, Levallois-Perret, France) and re-plating following initial culture setting. Under these permanent conditions, cells grew and were maintained as floating neurosphere-like colonies.

### AC133 labeling and flow cytometry

Glioma cells exposed to different oxygen tensions were collected and dissociated using Versene (Lonza). A total of 1.5×10^5^ cells were incubated with 5 μg/ml AC133 antibody (Miltenyi Biotech, Paris, France) or IgG1 isotype control (BD-Biosciences, Le Pont-de-Claix, France) for 1 h at 4°C in PBS containing 5% FBS and 0.02% sodium azide. Cells were then washed three times in PBS containing 5% FBS and 0.02% sodium azide, and incubated for 30 min at 4°C with FITC-conjugated goat anti-mouse IgG F(ab’)_2_ fragment polyclonal antibody (Dakocytomation, Trappes, France) at 20 μg/ml in PBS containing 5% FBS and 0.02% sodium azide. Following three more washes in PBS containing 5% FBS and 0.02% sodium azide, cells were re-suspended in PBS containing 2% formaldehyde and 0.02% sodium azide. A BD FACSCalibur™ fluorescent-activated flow cytometer and the BD CellQuest™ software (BD-Biosciences) were used in order to proceed to flow cytometry acquisition. Analysis was carried out using WinMDI 2.9 software (Scripps Institute, La Jolla, CA, USA).

### Treatment of human glioma cells with cobalt dichloride (CoCl_2_)

GlioA, GlioB, and GlioC human glioblastoma cells were dissociated in Versene (Lonza). They were then plated at 37.5×10^5^ cells per ml in the aforementioned media and incubated in the presence of vehicle alone (PBS) or 100–150 μM CoCl_2_ for 24 h at 37°C, 5% CO_2_ and 3% O_2_.

### shRNA knockdown

Glioblastoma cells were stably transfected using control transduction particles (SHC001V) or shRNA transduction particles expressing siRNA against HIF-1α (IDs: TRCN0000003810, TRCN0000003811 and TRCN0000010819), according to the manufacturer’s instructions (Mission^®^ pLKO.1-puro lentiviral particles, Sigma-Aldrich). Cells were seeded at 5×10^3^ in 96-well plates in supplemented neurobasal medium, and infected with a multiplicity of infection of 2. Puromycine (1 μg/ml, Sigma-Aldrich) selected infected cells.

### Q-PCR

Q-PCR analyses were carried out using a Chromo 4™ (Bio-Rad, Marnes-la-Coquette, France) and SYBR Green detection (iQ-SYBR Supermix, Bio-Rad). Primers were designed using Primer3 software (http://frodo.wi.mit.edu/primer3/). The ΔCt method was retained for quantification, and multiple genes were used for normalization, as previously described (27).

### Orthotopic xenograft assays

GlioA and GlioB human glioblastoma cells, grown at 3 or 21% O_2_, were dissociated in Versene, washed, and resuspended at 50,000 cells in 5 μl Eagle’s minimum essential medium (EMEM, Biowhittaker). SCID female mice (Charles River) were anesthetized using xylazine (50 μg/g) (Rompun^®^, Bayer, Puteaux, France) and Ketamine (10 μg/g) (Clorketam^®^, Vétoquinol, Lure, France). Stereotactic implantation of the 5 μl cell suspension was carried out into the right striatum using a Hamilton syringe and a 32-gauge needle at the following coordinates: 0.5 mm anterior from Bregma, 2 mm lateral from the saggital suture, and 3 mm below dura. Cells were injected progressively over 2.5 min, followed by 5 min of waiting, and progressive needle removal from brain over 6 min. MRI was used to monitor tumor growth. The Kaplan-Meier method was used to plot animal survival. Animal care was carried out in line with relevant European Community regulations (Official Journal of European Community L358 12/18/1986).

### Magnetic resonance imaging

Experiments were performed with a Bruker Avance DRX 300 (Bruker, Wiessembourg, France), equipped with a vertical super wide bore magnet and shielded gradient insert. The resonant circuit of the nuclear magnetic resonance (NMR) probe was a 38-mm diameter birdcage. Rectal temperature was maintained at 37°C by using a feedback-regulated heating pad. Brain lesion evolution was assessed using T2-weighted images obtained using a rapid acquisition with relaxation enhancement (RARE) (TR = 2000 ms; effective echo time = 31.7 ms; RARE factor = 8; FOV = 2.5 × 2.5 cm; matrix 128×128; nine contiguous slices of 1.2 mm, four averages). In order to improve tumor detection, FLAIR imaging was performed using a 600 ms inversion pulse prior to the RARE pattern, providing enough time to allow for the annulling of the normal parenchyma and therefore tumor detection.

### Immunohistochemistry

Brains from xenotransplanted mice were surgically removed, snap-frozen in isopentane cooled at −35°C with liquid nitrogen, and stored at −80°C before 10 μm transverse sections of anterior brain were made using a Cryocut 3000 (Leica, Rueil-Malmaison, France). After at least 24 h storage at −20°C and 30 min drying at room temperature, slides were fixed in −20°C cold methanol for 10 min. Sections were then blocked with 10% normal goat serum in PBS added with 4% bovine serum albumine for 30 min at room temperature. Primary antibodies against CD133 (clone AC133 and clone 293C3 both from Miltenyi Biotech) and the corresponding negative isotype controls (mouse IgG1κ and mouse IgG2b, both from BD Biosciences) were diluted in PBS containing 4% BSA and used at 5 μg/ml. They were applied overnight at 4°C. After washes in PBS, a secondary biotinylated goat anti-mouse IgG antibody (Vector Laboratories, Burlingame, USA) diluted in PBS containing 4% BSA was applied at 15 μg/ml for 45 min at room temperature. After additional washes in PBS, Alexa Fluor^®^ 488 streptavidine conjugates (Invitrogen, Cergy Pontoise, France) were applied in the dark at 4 μg/ml for 45 min. Finally, labeled sections were washed three times with PBS before mounting in fluorescent mounting medium from Dakocytomation. All slides were examined under an Axioskop-2 Zeiss fluorescence microscope (Le Pecq, France). Images were acquired through a Photometrics CoolSNAP ES camera equipped with a QImaging CRI Micro Color 2 RGB Liquid Crystal filter and by using the MetaVue™ imaging system (all from Roper Scientific, Evry, France).

### Statistical analysis

XLSTAT 2006 Version 2006.3 (Addinsoft Paris, France) was used for data analysis. Statistical significance for each experiment was determined by a Dunnett’s test. Alternatively, the Gehan-Wilcoxon and the Mann and Whitney non-parametric tests were used. The tests were considered as significant with p<0.05.

## Results

### Oxygen tension impacts the AC133 phenotype of human glioma cells in vitro

**Figure 1:**
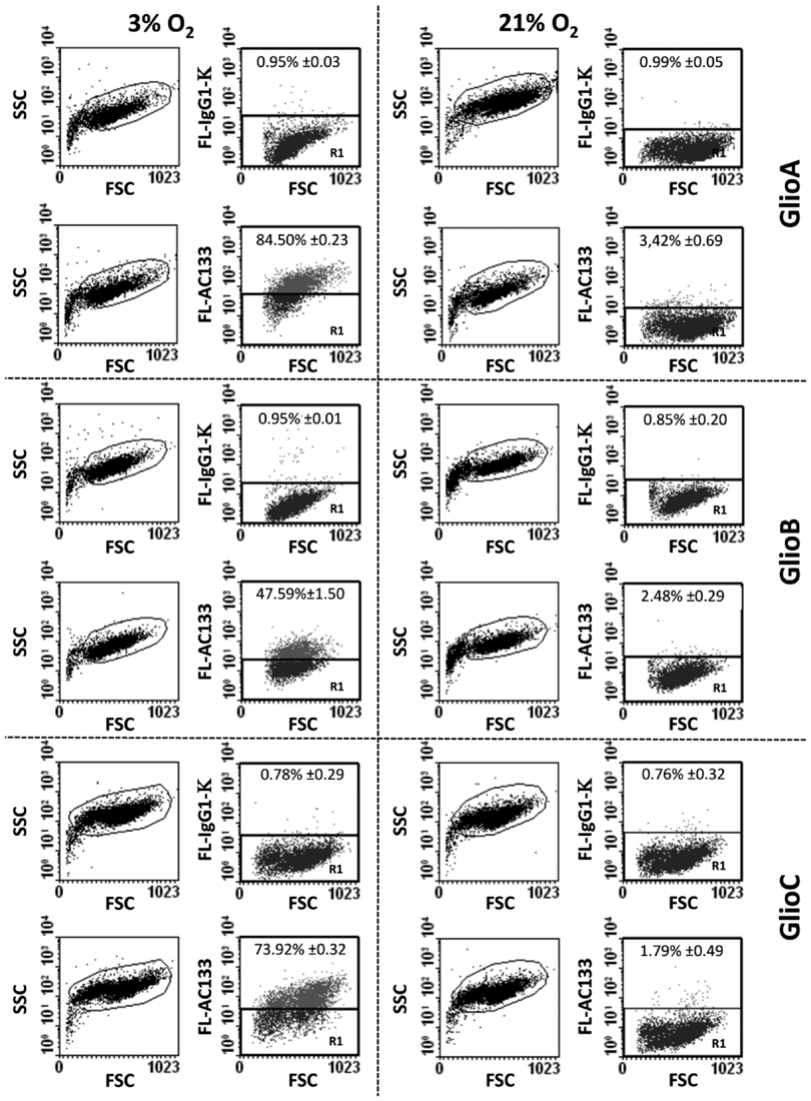
Greater percentage of AC133 positive cells are obtained in glioblastoma cells cultured at low pO_2_. GlioA, GlioB, and GlioC glioblastoma primary cells were cultured at 3 or 21% O_2_ and analyzed for AC133 expression using the anti-AC133 monoclonal antibody (AC133) or an IgG1κ isotype control (IgG1κ). The FITC fluorescence after application of the corresponding secondary antibody is expressed in geometric mean arbitrary units. FSC represented the forward scattering. Indicated percentages represent relative cell numbers that were up to the gated isotype control cells reported on each panel (gate R1). Dot plot profiles illustrate a representative experiment of at least a triplicate, at passage 21 (GlioA), passage 14 (GlioB) and passage 11 (GlioC) at 3 or 21% O_2_.

To determine the effect of oxygen pressure on AC133 expression in non-sorted primary human glioblastoma cells, GlioA, GlioB, and GlioC were cultured either at 3% O_2_ or 21% O_2_. High expression of AC133 was found in all cell lines when maintained at low oxygen tension (from initial suspensions to at least passage 30). As such, at matching cell passages flow cytometry analysis revealed that the percentage of AC133-positive cells was improved from 3 to 21% O_2_ condition (Fig. 1). Quantification of geomean fluorescence intensity further indicated a mean reduction of AC133 expression per cell up to 99% between 21 and 3% O_2_ (Table I).

### A role for HIF-1α in the regulation of AC133 expression

Having established a role for oxygen tension in the regulation of AC133, we next investigated whether HIF-1α a major transcription factor regulated by oxygen tension, was involved in this effect. HIF-1α has been described to be over-expressed in various cancers including gliomas (28). It heterodimerizes with constitutively expressed subunit HIF-1β to form HIF-1, a basic helix-loop-helix structure that regulates the transcription by specifically recognizing a short consensus HRE (hypoxia responsive element) sequence in the promoter of hypoxia responsive genes. HRE sequence is characterized by the presence of a consensus core CGTG found in all known HIF-1α-responsive promoters (29). A multiple sequence alignment ClustalW2 program revealed that the consensus core was present in all CD133 promoters from P1 to P5. Interestingly, the analysis showed that a sequence of 12 nucleotides present in P5 (known to be functional in stem cells) TACGTGCTCTGG-nucleotides 5416–542 matched perfectly with that present in the [+656/+667] HRE sequence of the human IGFBP-1 gene (30). Hence, this sequence represents a potential target for the binding of HIF-1 in glioblastoma cells.

**Figure 2:**
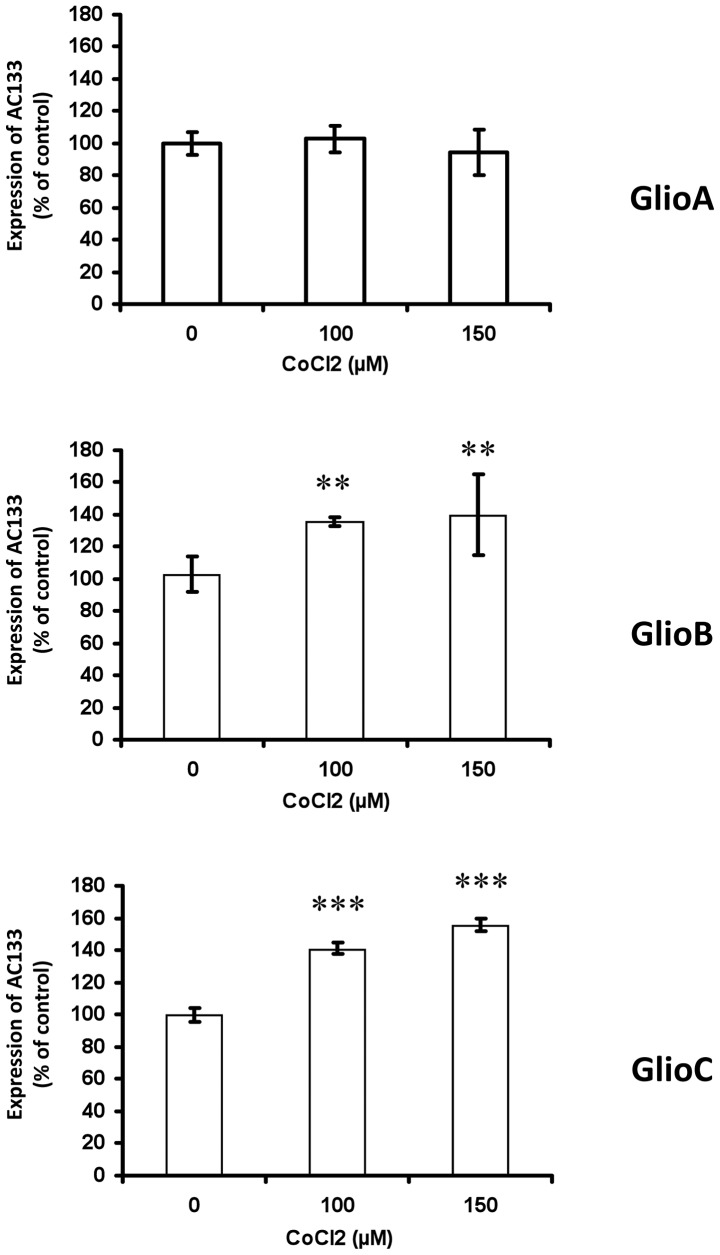
AC133 expression was up-regulated by CoCl_2_ treatment in glioblastoma cells. GlioA, GlioB, and GlioC primary human glioblastoma cells cultured at 3% O_2_ were incubated with 0, 100 and 150 μM CoCl_2_ for 24 h and then analyzed for AC133 expression by flow cytometry. Results are expressed as percentage of control, representing the geomean fluorescence intensity levels obtained after immunostaining of AC133 for cells treated with vehicle alone. Results represent mean ± SEM of three independent experiments. Dunnett’s test: ^**^p<0.01, ^***^p<0.001.

**Figure 3:**
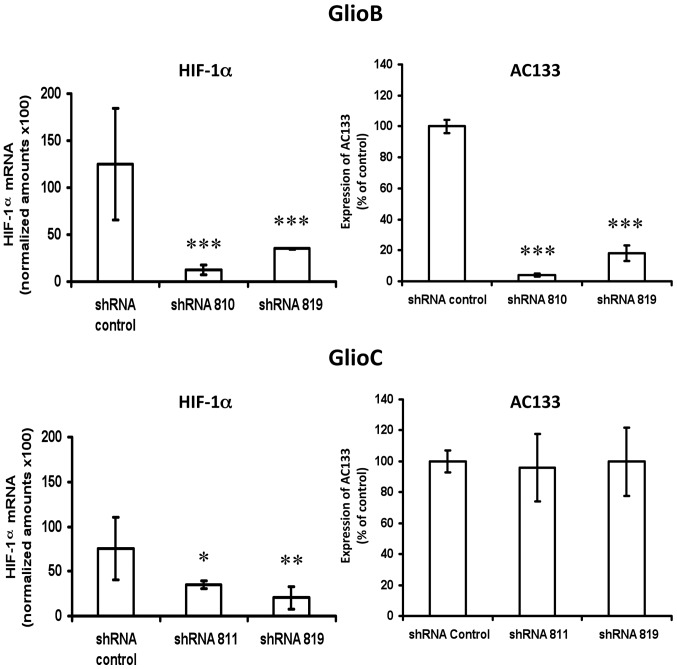
HIF-1α knockdown led to a reduced AC133 expression at 3% O_2_ in GlioB cells but not in GlioC. Left panels show that transfection of GlioB and GlioC cells with specific shRNA against HIF-1α RNA (810, 811 or 819) in HIF-1α mRNA levels when compared with irrelevant shRNA control as determined by RT-qPCR. Results are expressed in normalized amounts as indicated in Materials and methods. Right panels indicate that while knockdown of HIF-1α resulted in dramatic inhibition of AC133 expression in GlioB as assessed by flow cytometry, no effect was observed in GlioC. Results are expressed as percentage of control, representing the geomean fluorescence intensity levels obtained after immunostaining of AC133 for cells treated with shRNA control. They also represent mean ± SEM of three independent experiments. Dunnett’s test: ^*^p<0.05, ^**^p<0.01, ^***^p<0.001.

To determine the potential influence of HIF on regulating the expression of AC133, cobalt chloride (CoCl_2_), which inhibits the degradation of HIF (31), and the shRNA knockdown strategy against HIF-1α were used. As HIF-1α stabilization has been shown to increase from moderate to severe hypoxia while not induced under ambient air (32), in order to try getting its level maximal, the hypoxia-mimetic CoCl_2_ was used already from the 3% O_2_ condition. When GlioA cells were incubated for 24 h with 100 or 150 μM of CoCl_2_ in low pO_2_ conditions, no significant change was observed in AC133 expression as compared to control culture (Fig. 2). In contrast, CoCl_2_ treatment increased the expression of AC133 in GlioB and GlioC (+36–41% for GlioB and +41–56% for GlioC) (Fig. 2). We further address the impact of HIF-1α inhibition on CoCl_2_ responding glioblastoma cell types. Transcriptional down-regulation of HIF-1α mRNA with a lentiviral shRNA-based system performed on GlioB (knockdown efficency of ~80% and GlioC (knockdown efficiency of ~65% (Fig. 3 left panels) was associated with a 80–90% reduction in AC133 expression for GlioB, but had no impact on AC133 expression for GlioC (Fig. 3 right panels).

### Human glioma cells exposed to different oxygen tensions in vitro do not behave equally following orthotopic transplantation in immunodepleted mice

**Figure 4:**
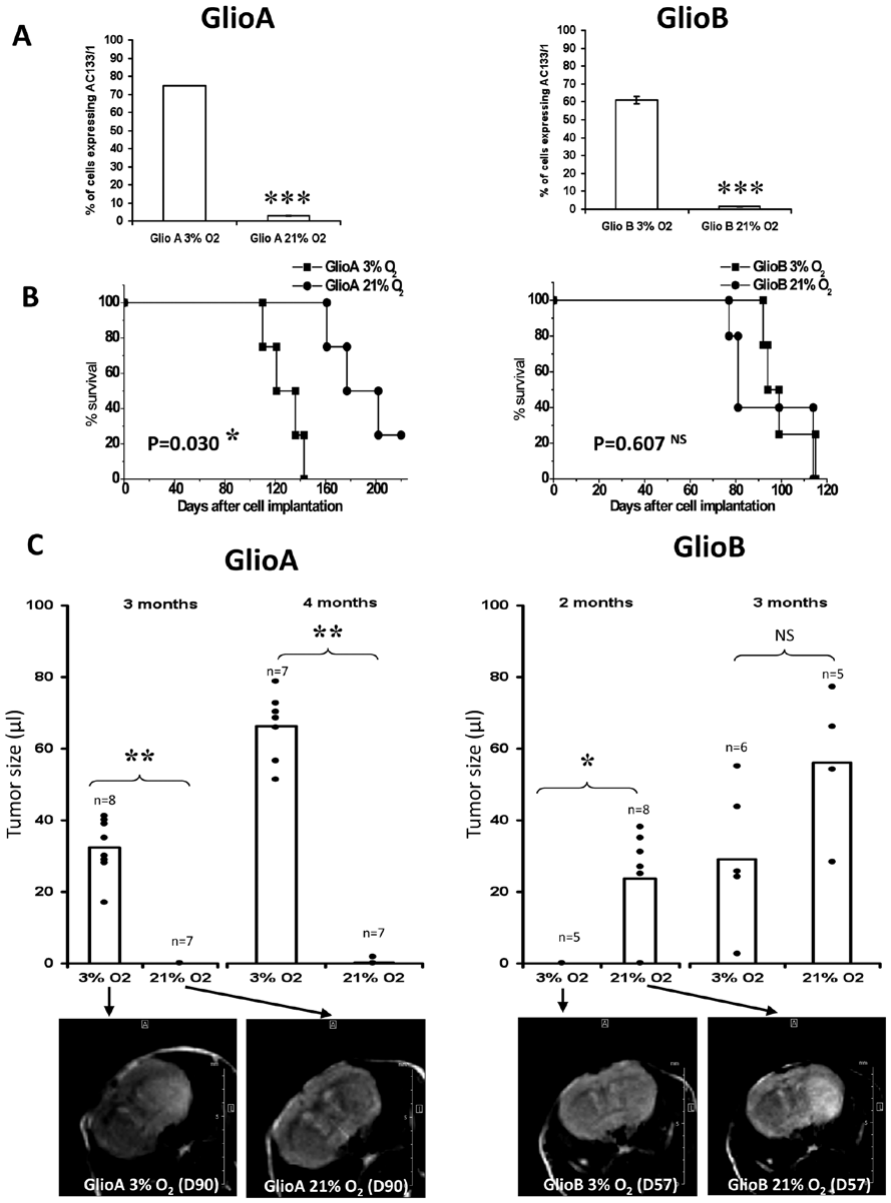
Culturing glioblastoma primary cells at 3 or 21% O_2_ resulted in distinct cell behavior *in vivo*. (A) Flow cytometry analysis of AC133 expression before implantation confirms that GlioA and GlioB cells cultured at 3% O_2_ expressed more AC133 than did cells cultured at 21% O_2_. (B) Kaplan-Meier survival curves following implantation of 50,000 cells into the right striatum of female SCID mice revealed that cells cultured at 3% O_2_ were more aggressive than their 21% O_2_ counterparts for GlioA but nor for GlioB. Gehan-Wilcoxon test: NS, not significant; ^*^p<0.05. (C) Measurement of tumor sizes assessed by MRI analysis and corresponding representative MRI images for mice implanted with GlioA or GlioB. Note that although tumors obtained with GlioB grown at 3% O_2_ were not detectable at 2 months after brain implantation, they close in on their 21% O_2_ counterparts 1 month later, thus revealing distinct growth modalities and density contrast at a given point in time for cells maintained in culture at 3 versus 21% O_2_. Mann-Whitney U test: NS, not significant; ^*^p<0.05; ^**^p<0.01.

Having established a role for oxygen tension and HIF-1α in regulating AC133 *in vitro*, we wished to further address whether tumor development and AC133 expression were affected by the expansion of human glioblastoma cells under different oxygen tension culture conditions. For this purpose, we focused on the cell types for which tumors were detected through MRI monitoring within 3 months after stereotactic injection of glioblastoma cells in the right striatum of immunodepleted mice, namely GlioA and GlioB (Fig. 4). Kaplan-Meyer curves shown in Fig. 4A revealed that GlioA cell cultures at 3% O_2_ were more aggressive than GlioA cells cultured at 21% O_2_. In contrast, no significant differences in Kaplan-Meier curves were observed on GlioB. However, tumors caused by the implantation of GlioA cultured at 3% O_2_ prior to injection were detected earlier than the tumors arising from GlioA cultured under 21% O_2_ (Fig. 4B). Indeed, mice injected with the cells cultured at 3% developed a detectable tumor within 3 months post-injection (average tumor size 32±8 μl (n=8)], whereas a similar size was observed 5 months post-injection of GlioA cells initially cultured at 21% O_2_.

Interestingly, although no differences were observed on Kaplan-Meier curves, GlioB cultured at 21 vs. 3% O_2_ appeared to differ on MRI images. When GlioB cells were cultured *in vitro* at 21% O_2_ prior injection, brain tumor occurred within 2 months [average tumor size 23±13 μl (n=8)], whereas injection of cells cultured at 3% O_2_ reached such a size after 3 months [average tumor size 34 ± 21 μl (n=6)] (Fig. 4B). Examined together, the data indicate that culture conditions are likely to exhibit a real impact on tumor aggressiveness *in vivo*, underlying the fact that the choice of culture parameters can modulate cell behavior *in vivo*.

### Extinction of AC133 expression of human glioma cells exposed to low oxygen tension in vitro prevents in vivo re-expression after orthotopic transplantation in immunodepleted mice

**Figure 5:**
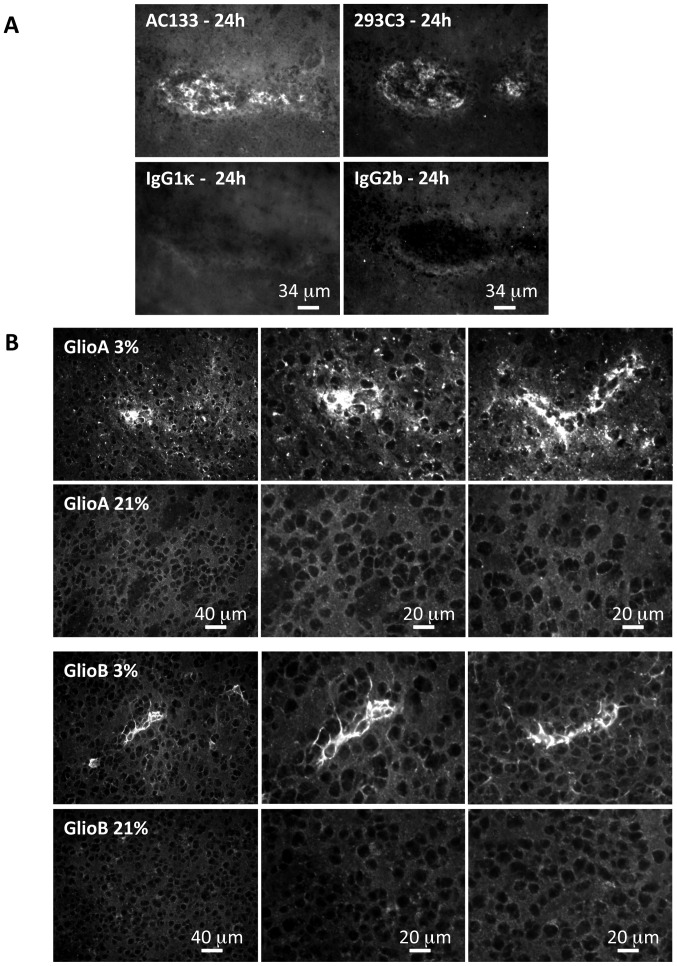
AC133 was not re-expressed *in vivo* in glioblastoma tumors obtained from cells cultured at 21% O_2_ while maintained in tumors derived from cells grown at 3% O_2_. (A) Validation of the immunohistochemical detection of AC133. Analysis was initially performed using AC133 and 293C3 antibodies as well as respective isotype controls (IgG1κ and IgG2b) on mouse brain adjacent cryosections 24 h after implantation of 50,000 GlioA glioblastoma cells grown at 3% O_2_ known to express AC133 *in vitro*. Note common staining characteristics between AC133 and 293C3. Also note the absence of background when using isotype controls at the same concentrations. (B) Although AC133 was detected in discrete areas of both GlioA and GlioB tumors derived from cells grown at 3% O_2_, it was not found on brain tumors derived from cells grown at 21% O_2_.

In order to address human AC133 expression in mice with tumor growth, a study was carried out on mice 24 h post-injection of AC133 positive cells to validate AC133 immunohistochemical detection using the AC133 antibody or 293C3 antibody, both recognizing two different human epitopes of the CD133 protein (Fig. 5A).

Applying this technique to brain tumors collected at the end point of the experiment revealed that when the injected cells were initially cultured at 3% O_2_, AC133 was still detected, and this for both GlioA and GlioB cells (Fig. 5B). However, AC133 was detected in limited clusters within the tumor, suggesting that not all the tumor cells had kept the AC133 phenotype. The same approach on tumors arising from cells cultured at 21% O_2_ did not reveal any AC133 expression (Fig. 5B), indicating that neither GlioA nor GlioB cells grown in 21% O_2_ before injection gave rise to AC133 cells *in vivo*.

## Discussion

### Consequences of the 21% standard pO_2_ culture condition on glioblastoma phenotypes

To address cancer cell behavior *in vivo* and *in vitro*, cancer cell cultures are generally performed under 5% CO_2_ combined to classical atmospheric conditions of approximately 21% O_2_ (160 mm Hg). However, pO_2_ values do not exceed 12% O_2_ (95 mm Hg) in the blood and vary from 1 to 5% (6–34 mm Hg) in normal tissues including the brain (33). Moreover, a characteristic feature of advanced solid tumors is to display hypoxic tissue areas (pO_2_ ≤0.4% or 2.5 mm Hg) due to insufficient vascularization, oversize tumor mass, and necrosis (34). Thus, an atmosphere containing 21% O_2_ should be physio-logically considered hyperoxic. In the present study, although glioblastoma cells were cultured as three-dimension neurospheres, a condition that lowers pO_2_ due to the gradient of O_2_ diffusion from the external to the inner part of the spheres, our data demonstrated that *in vitro* pO_2_ ranges obtained at 3 versus 21% O_2_ resulted in distinct cell behavior *in vivo*. Tumor aggressiveness was higher for GlioA when cultured at 3 versus 21% O_2_. MRI detection of GlioB grown at 3% was delayed when compared to GlioB grown at 21%, while ultimately giving rise to similar adverse clinical effects. Moreover, AC133, typically found on fresh human glioblastoma biopsy specimens (21) or on short-term primary glioblastoma cultures [(3,13); our study]; was maintained after expansion *in vitro* at 3% O_2_ while lost at 21% O_2_ and not re-expressed after cell implantation *in vivo*. These combined findings stressed that pO_2_ values obtained at 3% O_2_ preserve better the AC133 phenotype of glioblastoma cells than do pO_2_ values obtained through the standard O_2_ atmospheric tension. Our results confirmed, therefore, that a low pO_2_ (≤3% O_2_ or 24 mm Hg) should be considered a basic condition to study glioblastoma cell behavior in their current microenvironment. As such, the fact that pO_2_ irreversibly changes the phenotype of glioblastoma cell populations is also reminiscent of the effects of serum and laminin on gene expression profiles, expression of stem cell makers, and glioma invasiveness (3,35,36). As variations of pH, the traditional 21% O_2_ represents a new environmental stress for glioblastoma cells that inevitably triggers alterations of their differentiation, genetic and epigenetic status, and survival. Considering tumor heterogeneity, selection of glioblastoma cell clones will therefore be different at 3 and 21% O_2_. As such, low oxygen tension is often perceived as an obstacle for chemo- and radiotherapy due to the induction of several resistance genes (13,36), DNA repair or methylation (22), miRNA expression (37), and maintenance of stemness (38). Conversely, high oxygen tension represents an oxidative stress that may be associated with the selection of cells that are well-equipped for reactive oxygen species detoxification (39).

### Is AC133 a marker of BTSC non-chronic exposure to high oxygen tension?

AC133 has initially been described as a marker of hematopoietic stem cells (9,11), while then associated with embryonic stem cells (40) and a variety of somatic stem cells (41). AC133 was also recognized as a putative cancer stem cell marker in blood, brain, colon, prostate, lung, breast, liver, and skin cancers (12,41). Although the BTSC hypothesis was strongly supported by recent data (4,42,43), the idea of a responsibility of cancer stem cells in glioblastoma development remains to be documented (44) and does not exclude the role of clonal selection (45). We emphasize that if the hypothesis of brain cancer initiating cells is correct, the loss of AC133 does not preclude their occurrence. We did establish that GlioA and GlioB that do not contain high AC133 expressing cells when cultured at 21% O_2_ self-renew *in vitro* and do form tumors *in vivo*. The data give further significance to the originally established unique ability of immunosorted AC133-positive cells to form brain tumors (2,3,13,46) and corroborate the fact that AC133-negative cells are also capable of doing so (47,48). Thus, high AC133 expression is not a marker of every cancer initiating cells within brain tumors.

Sorted AC133-positive cells have been described as more aggressive than their AC133-negative counterparts (2,3,13). Our data proved that when considering the full cancer cell population, the major reduction in AC133 expression at high versus low pO_2_ (87.6%, GlioB, Table I) as well as in AC133 positive cell numbers (from 47.59 to 2.48%, GlioB, Fig. 1) allows for the development of tumors that are similarly aggressive. Thus, AC133 does not appear to be a general marker of tumor aggressiveness.

Low oxygen tension was associated with the stem cell-like properties of AC133-positive glioblastoma cells (26). As we have confirmed here that this also resulted in high levels of AC133, one might assume that AC133 expression constitutes a witness of low oxygen tension. The presence of putative HRE in Prominin-1 promoters combined with the modulation of AC133 expression by CoCl_2_ treatment and HIF-1α shRNA knockdown supported this assertion. Previous data obtained with siRNA against HIF-1α (49), or instead with an oxygen stable HIF-1α construct (50), also corroborated this, with a significant role for HIF-1α. Interestingly, in contrast to what happen in glioblastoma, in gastric, colorectal and lung cancer cell lines Matsumoto *et al* established an inverse correlation between HIF-1α and CD133 expression thus indicating tissue specificities for the regulation of CD133 by HIF-1α (51). However, CoCl_2_ did not induce AC133 in GlioA O_2_, and HIF-1α shRNAs were not able to reduce AC133 expression in GlioC. Although constitutive expression of AC133 might be maximal in GlioA, and HIF-2 is likely to compensate for the loss of HIF-1α in GlioC (21,52), HIF-independent pathways may be involved in the AC133 regulation by hypoxia. A variety of these recognizable cell signals that translate to environmental O_2_ changes have already been described, including: reactive oxygen species (53), thiol-based sensors (53), the transcriptional co-activator PGC-1α (54), or mTOR inhibition via the AMPK/TSC2/Rheb pathway (55). Regardless of the signaling pathway involved in regulating AC133 by pO_2_, we have established in our study that the loss of AC133 at 21% O_2_ *in vitro* (data not shown) and *in vivo* following glioma cell implantation in mouse brains was irreversible. This lack of re-expression of AC133 therefore supported the fact that AC133 is not a genuine marker of hypoxia in glioblastoma. Indeed, low pO_2_ commonly involved in glioblastoma growth and aggressiveness (23,24) should be present within GlioA and GlioB tumors, which was supported by a reduced vascularization observed using CD31 labeling (data not shown). One-way regulation of AC133 by pO_2_ might be explained by the acquisition of a new pattern of transcriptional activators or a new DNA methylation status of glioblastoma cells at 21% (56,57).

**Figure 6:**
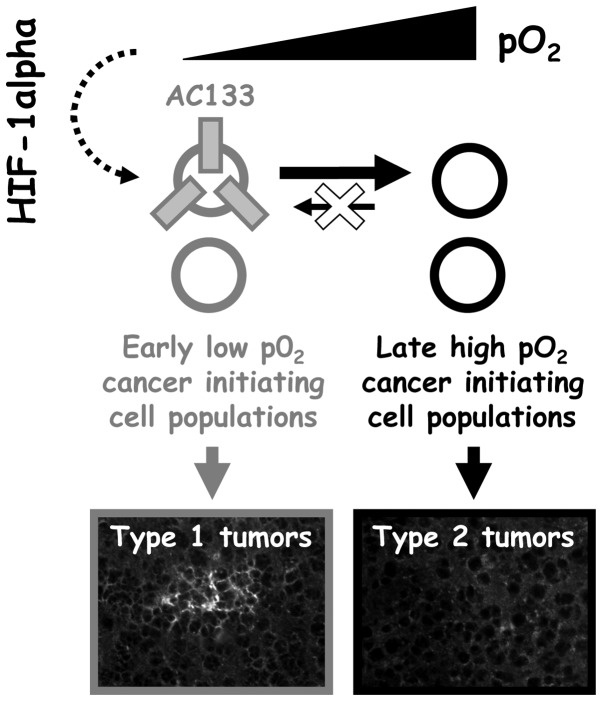
Model defining the relationships between the environmental pO_2_ and the AC133 phenotype. During gliomagenesis, high oxygen tension may be a component of the cancer initiating cell niche that drives an early-to-late cancer initiating cell switch. In this situation, the irreversible loss of AC133 expression would be perceived as a witness of glioblastoma cell maturation. Instead of being a marker of tumorigenicity, aggressiveness, or oxygen supply, AC133 notably regulated by HIF-1α therefore represents a hallmark of glioblastoma cell non-exposure to high oxygen tension. Thus, early cancer cell populations that contain AC133-positive cells formed type 1 tumors that continue to express AC133, while late AC133-negative cancer cell developed type 2 tumors which did not re-express AC133.

As AC133 does not attest to the glioma cell capability of forming tumors or to glioblastoma aggressiveness or low oxygen tension, we propose that it represents a witness of glioblastoma cell non-exposure to high oxygen tension. The presence of AC133 positive glioblastoma cell populations that have also been established at ambient oxygen setting could be explain in this context by creation of hypoxic gradients within the growing glioma spheres (58). This fact would be attenuated by chronic exposure of cells to high oxygen tension through sequential dissociation and re-plating. Hence, similarly to developmental cues that lead to irreversible maturation of early-to-late neural stem cell differentiation during development such as FGF (59), high oxygen tension may represent a component of the BTSC niche that drives an early-to-late BTSC switch during gliomagenesis. If EGF receptor expression represents a witness of the acquired phenotype for neural stem cell maturation, loss of AC133 would be a witness of BTSC maturation. To support this assertion, the loss of AC133 expression has been associated with cancer stem cell differentiation in glioblastoma (60) and in colon cancer (61). Moreover, use of glioma cell differentiation factors such as retinoic acid lead to down-regulation of AC133 expression (62). In addition, transdifferentiation of tumor cells into vessel formation was recently associated with stemness phenotype and hypoxia in glioblastoma (63). Thus, irreversible AC133-loss may also have an impact on this epithelial to mesenchymal transition reciprocally. Two types of tumors could therefore be obtained from non-sorted human glioblastoma cells expanded *in vitro*: type 1 tumors obtained from 3% O_2_-expanded cells (expressing AC133) and type 2 tumors obtained from 21% O_2_-expanded cells (no AC133 expression) (Fig. 6).

In conclusion, our present study underlines that non-physiological oxygen tension alters subsequent *in vitro* expansion and *in vivo* development of non-sorted human glioblastoma cells. With the preservation of AC133 expression, which can result from the prevention of AC133-positive cell death or from continuous prominin-1 gene expression, the 3% O_2_ expansion condition mirrors much the biological reality. Thus, the timing of environmental pO_2_ variations likely reflects a changing pattern of plasma membrane protein expression during glioblastoma growth that is associated with cell heterogeneity and resistance. The fact AC133 was here associated with an early glioblastoma phenotype suggests that identification of downstream cancer initiating cell markers as well as evaluation of relative anticancer drug sensitivity of type I and type II tumors (Fig. 6) would also be helpful in the development of anti-glioblastoma strategies.

## Figures and Tables

**Table I:** Glioblastoma cells cultured at low pO_2_ expressed improve levels of AC133 than those cultured at high pO_2_.^a^

	GMFI at 3% O_2_ (arbitrary units)	GMFI at 21% O_2_ (arbitrary units)	Mean variation of AC133 expression per cell
GlioA (passage 21)	77.42±0.15	1.03±0.25	−98.7%
GlioB (passage 14)	16.70±0.56	2.08±0.15	−87.6%
GlioC (passage 11)	52.07±1.59	0.65±0.17	−98.7%

a GlioA, GlioB, and GlioC glioblastoma primary cells were cultured at 3 or 21% O_2_ and analyzed for AC133 expression using the anti-AC133 monoclonal antibody (AC133) or an IgG1κ isotype control (IgG1κ).

FITC fluorescence revealed after application of the corresponding secondary antibody was expressed in geometric mean fluorescence intensity (GMFI) arbitrary units. Mean variation of AC133 expression per cell was calculated according to GMFI obtained at 3 versus 21% O_2_.
